# Bioinformatics Analysis Reveals the Altered Gene Expression of Patients with Postmenopausal Osteoporosis Using Liuweidihuang Pills Treatment

**DOI:** 10.1155/2019/1907906

**Published:** 2019-01-27

**Authors:** Rui Gong, Shan Ren, Menghui Chen, Yanli Wang, Guoliang Zhang, Lijuan Shi, Cuizhao Zhang, Ruihong Su, Yukun Li

**Affiliations:** ^1^Hebei Medical University Endocrine Research Institute, China; ^2^Department of ICU, Hebei General Hospital, China; ^3^Department of Cardiothoracic Surgery, The Third Hospital of Shijiazhuang, China; ^4^Obstetrics and Gynecology, The Third Hospital of Shijiazhuang, China; ^5^Department of Endocrinology, The Third Hospital of Shijiazhuang, China; ^6^Department of Laboratory, The Third Hospital of Shijiazhuang, China; ^7^Department of Endocrinology, The Third Affiliated Hospital of Hebei Medical University, China

## Abstract

Postmenopausal osteoporosis (PMOP), as well as its associated increased risk for fragility fracture, is one of the most disabling consequences of aging in women. This present study aimed to identify candidate genes that involve pathogenesis of PMOP and the therapeutic mechanism of Liuweidihuang (LWDH) pills on PMOP. We integrated microarray datasets of PMOP derived from the Gene Expression Omnibus (GEO) to screen differentially expressed genes (DEGs) between PMOP and normal controls as well as patients with PMOP and patients after treatment of LWDH pills. GO and KEGG enrichment analysis for DEGs were performed. The shared DEGs, associated with both the pathogenesis of PMOP and the therapeutic mechanism of LWDH, were further analyzed by protein-protein interaction (PPI) network. Quantitative real-time polymerase chain reaction (qRT-PCR) was performed to verify the DEGs obtained by our integrated analysis. Compared with normal controls, 1732 DEGs in PMOP were obtained with* p*<0.05. According to the qRT-PCR results, expression of ATF2, FBXW7, RDX, and RBBP4 was consistent with that in our integrated analysis, generally. GO and KEGG enrichment analysis showed that those DEGs were significantly enriched in regulation of transcription, DNA-dependent, cytoplasm, protein binding, and MAPK signaling pathway. A total of 58 shared DEGs in PMOP versus normal control and in patients with PMOP versus patients after LWDH treatment were identified, which had opposite expression trend in these two comparisons. In the PPI network, CSNK2A1, ATF2, and FBXW7 were three hub proteins. Three genes including ATF2, FBXW7, and RDX were speculated to be therapeutic targets of LWDH for PMOP based on BATMAN-TCM database. We speculated that three genes of ATF2, FBXW7, and RDX may play crucial roles in both pathogenesis of PMOP and therapeutic mechanism of LWDH on PMOP. Our results may provide clues for the molecular pathogenesis of PMOP and offer new possibilities for treatment of PMOP.

## 1. Introduction

Postmenopausal osteoporosis (PMOP) often occurs due to the simultaneous interaction of independent predisposing factors, including aging and continuous calcium loss [1]. In the developed country, 9-38% of females are affected by PMOP. It is estimated that osteoporosis-associated fractures affect one-third of women in their lifetime, which is a public health concern [2]. Recently, both in animal models and in humans that were affected by osteoporosis newly, evidences of the relationship between immune system and bone have been accumulated. Estrogen is a well-known regulator of the immune system and T-cell functions. Bone loss induced by estrogen deficiency in menopause is a complex effect of a multitude of pathways and cytokines working in a cooperative fashion to regulate osteoclastogenesis and osteoblastogenesis [3–6]. Some hypotheses have been done by previous literature data. Recent studies have shown that obesity and osteoporosis share common genetic and environmental factors. Wnt family members and Wnt inhibitor molecules were involved not only in the regulation of differentiation of multipotent mesenchymal stem cells into osteoblasts and adipocytes but also in the coordination of the switch toward osteo- or adipogenesis fate within bone marrow[7].

Microarray studies have been used to explore the pathogenesis of PMOP. Expression profiling with larger sample size could be obtained by integrated microarrays analysis, which contributes to identifying more accurately differentially expressed genes (DEGs) of PMOP than an individual microarray. In this study, we performed an integrated analysis of five gene expression datasets to identify the DEGs between PMOP and normal controls and explore the molecular mechanism of PMOP. Functional annotation and PPI network construction were performed to explore the biological functions of these DEGs.

Various medications, including alendronate, etidronate, risedronate, and strontium ranelate, have been employed to prevent osteoporotic fragility fractures in patients with PMOP [8]. MF Faienza et al. demonstrated that denosumab and romosozumab have promise in the treatment of osteoporosis and their different mechanisms of action compared to existing antiosteoporotic drugs may permit alternative strategies for osteoporosis treatment down the line [9]. In recent years, research of traditional Chinese medicine has confirmed that incidence of osteoporosis was closely associated with kidney. Tonics for kidney, such as Liuweidihuang (LWDH) pills, were demonstrated to exert an effect on diabetes mellitus [10], breast carcinoma [11], and protection of testes in mice [12]. However, few studies were focused on the effect of LWDH on human osteoporosis. In the paper of Manyu Li [13], it is evaluated the effects of morroniside and loganin isolated from LWDH pills on the proliferation, differentiation, and apoptosis of MC3T3-E1 (mouse embryonic osteoblast precursor cells). Morroniside and loganin were found to promote the differentiation of osteoblast precursor cells and inhibit their apoptosis, thereby reducing bone resorption. Ji-rong Ge et al. found that CLCF1 is an important gene associated with Shen-yin deficiency of PMOP and the therapeutic effects of LWDH may be mediated by upregulated expression of CLCF1 and activation of the JAK/STAT signaling pathway [14]. In light of the potent curative effect of LWDH, exploring the candidate genes associated with both pathogenesis of PMOP and therapeutic mechanism of LWDH on PMOP could provide clues for the mechanism of PMOP and development of novel therapeutic strategies for PMOP.

## 2. Materials and Methods

### 2.1. Eligible Gene Expression Profiles of PMOP

We selected gene expression datasets of PMOP on the Gene Expression Omnibus database (GEO, http://www.ncbi.nlm.nih.gov/geo) which is the largest database of high-throughput gene expression data [15]. Search keywords in PMOP versus normal control were “postmenopausal osteoporosis” [MeSH Terms] OR “postmenopausal osteoporosis” [All Fields]) AND “gse” [Filter]. Datasets that meet the following criteria will be included in our study. (1) The selected dataset must be genome-wide mRNA transcriptome data. (2) These data are derived from postmenopausal osteoporosis patients or blood samples (without drug stimulation or transfection) of patients with osteoporosis before and after LWDH pills treatment was considered. (3) Five sets of high-throughput mRNAs were screened by the standardization or original data set.

### 2.2. Identification of DEGs Associated with PMOP or LWDH Treatment

Firstly, we analyzed all the selected databases individually. Then, to minimize the heterogeneity among different datasets enrolled in integrated analysis, normalization and log2 transformation were performed for the raw data. Finally, an R package, metaMA package, was used to combine data from multiple microarray datasets. Individual p-values were calculated and multiple comparison correction FDR was obtained by using Benjamini and Hochberg method. Genes with FDR < 0.05 were screened out and regarded as DEGs [16].

### 2.3. qRT-PCR Confirmation for PMOP

Based on the results of high-throughput transcriptome data integration analysis, four differentially expressed genes were screened as candidate genes (PMOP versus normal control); blood samples (6 cases) from PMOP patients and healthy individuals were collected. Six blood samples were frozen at 80°C within 2 hours after blood withdrawal. After thawing the frozen samples at room temperature, 1 ml of the sample was used to perform RNA isolation with Trizol reagent (Invitrogen, China) according to the manufacturer's instructions. By using SuperScript® III Reverse Transcriptase (Invitrogen, China), we generated cDNA from 1*μ*g extracted RNA. With Power SYBR® Green PCR Master Mix (Applied Biosystems, USA), we performed quantitative PCR in an ABI 7500 real-time PCR system. Relative gene expression was analyzed using 2^−ΔΔCt^ method. The human GAPDH gene was used as endogenous control for mRNA expression in analysis. The sequence of the primers used in RT PCR experiment was shown in Supplementary Table 1.

### 2.4. Functional Annotation of DEGs in PMOP and Common DEGs

To identify the function and the potential pathway of DEGs in PMOP and 58 common DEGs, Gene Ontology (GO) classification (molecular functions, biological processes, and cellular component) and Kyoto Encyclopedia of Genes and Genomes (KEGG) pathway enrichment were performed by using the online software GeneCodis (http://genecodis.cnb.csic.es/analysis) [17]. FDR < 0.05 was defined as the criteria of statistical significance. Fifty-eight genes were subjected to GO enrichment and KEGG enrichment analysis using the R language (GSEABase package), P-value < 0.01.

### 2.5. Protein-Protein Interaction (PPI) Network Construction

To further research the biological functions of 58 common DEGs, we constructed the PPI network by using Biological General Repository for Interaction Datasets (BioGRID) (http://thebiogrid.org/) and Cytoscape [18]. Based on the existing protein interaction with opposite effects on PMOP versus normal control and LWDH data of BioGRID database, Cytoscape was used to search for common and regulatory genes treatment versus PMOP. After removing the two nondifferentially expressed genes, the protein network interaction map was drawn. The network consists of nodes and edges. The nodes in the network represented the proteins and the lines represented the interactions between protein [19].

### 2.6. Target Prediction of LWDH in BATMAN-TCM Database

To define the key targets of LWDH, we retrieved BATMAN-TCM database, which is the first online Bioinformatics Analysis Tool for molecular mechanism of Traditional Chinese Medicine (http://bionet.ncpsb.org/batman-tcm/) [20]. LWDH was composed of five herbs, such as ZE XIE, SHAN YAO, MU DAN PI, SHU DI HUANG, JIU YU ROU, and FU LING. We input the herb list denoted by Pinyin name of ‘ZE XIE, SHAN YAO, MU DAN PI, SHU DI HUANG, JIU YU ROU, and FU LING' with the following default parameters: predicted candidate targets (including known targets) with scores not smaller than score cutoff = 20 for each ingredient are presented and used for further bioinformatics analyses. The targets of Liuweidihuang were then obtained by a combination of the targets of ZE XIE, SHAN YAO, MU DAN PI, SHU DI HUANG, JIU YU ROU, and FU LING.

## 3. Results

### 3.1. Differential Expression Analysis of DEGs in PMOP

**Figure 1:**
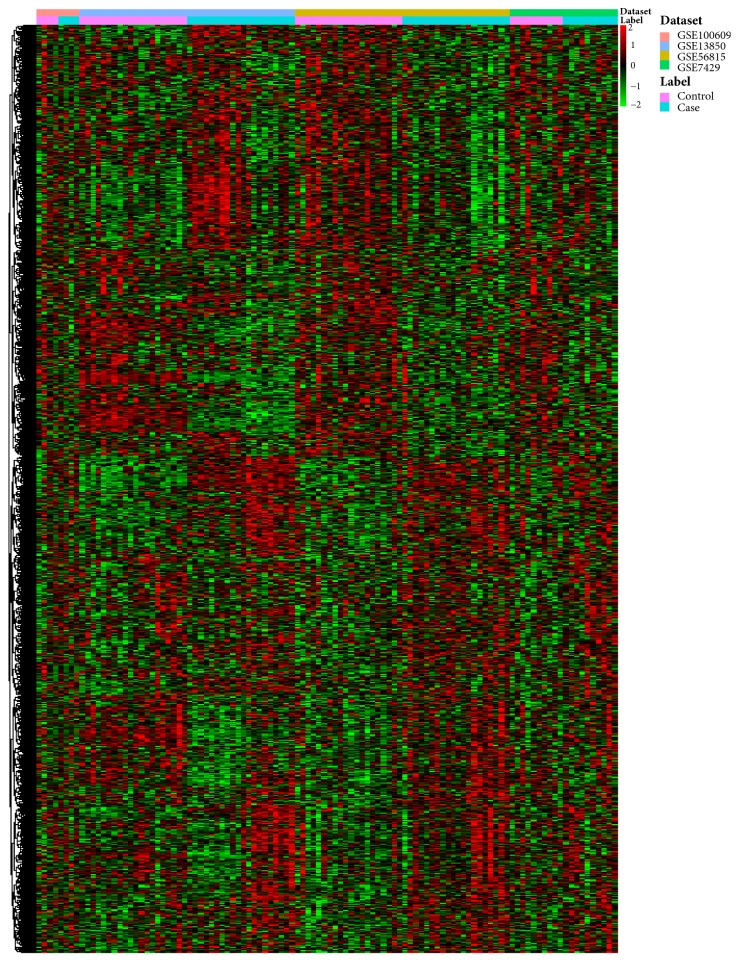
**The heatmap of DEGs in PMOP versus normal control**. Row and column were used to represent DEGs and datasets. The color scale represented the expression levels of DEGs.

**Figure 2:**
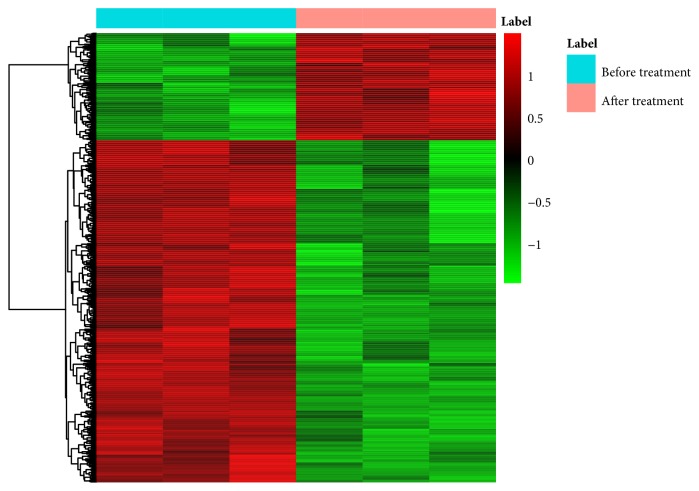
**The heatmap of DEGs in LWDH treatment versus PMOP**. Row and column were used to represent DEGs and datasets. The color scale represented the expression levels of DEGs.

Four gene expression microarray datasets (GSE100609, GSE56815, GSE13850, and GSE7429) and a microarray dataset associated with therapeutic mechanism of LWDH on PMOP (GSE57273) were enrolled in our study. The clinical features of the enrolled subjects were shown in Supplementary Table 2. In total, the ratio of PMOP versus normal control was 54:60. Compared with the normal controls, 1732 DEGs in PMOP were obtained with* p*<0.05; among which, 918 genes were upregulated and 814 genes were downregulated. The hierarchical clustering heatmaps of DEGs in PMOP versus normal control were shown in Figure 1. Likewise, there were 1350 DEGs (322 upregulated and 1027 downregulated) in patients with PMOP versus patients after LWDH treatment. The hierarchical clustering heatmaps of DEGs in patients with PMOP versus patients after LWDH treatment were shown in Figure 2.

### 3.2. Functional Annotation of DEGs in PMOP

**Figure 3:**
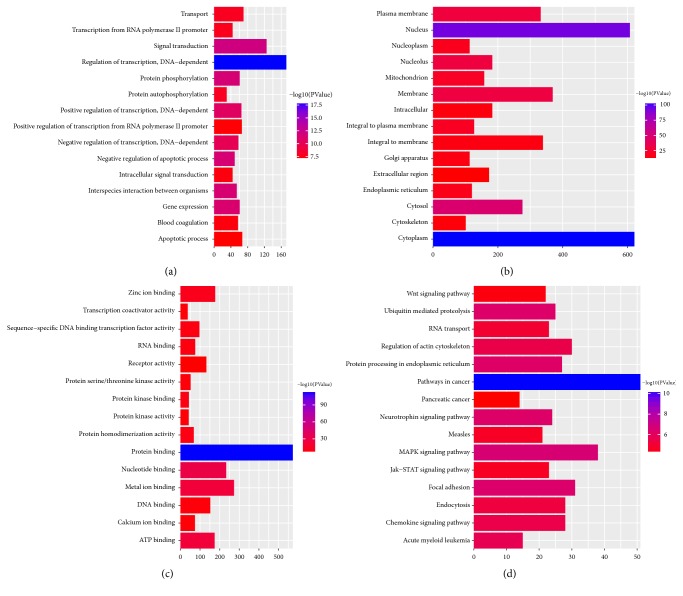
**Go and KEGG functional enrichment of DEGs in PMOP**. (a) Biological progress; (b) cellular component; (c) molecular function; and (d) KEGG functional enrichment. The y-axis represented GO and KEGG terms and the x-axis shows represented counts of DEmRNAs enriched in GO and KEGG terms.

In Figure 3, GO and KEGG enrichment analysis showed that DEGs were significantly enriched in the following terms. Biological processes: regulation of transcription, DNA-dependent (*p*=4.31E-22), and signal transduction (*p*=4.44E-16). Cell component: cytoplasm (*p*=4.14E-108) and nucleus (*p*=1.06E-95). Molecular function: protein binding (*p*=4.19E-115) and metal ion binding (*p*=9.37E-27). KEGG pathway enrichment results showed that DEGs were enriched in the Osteoclast differentiation (*p*=0.00931908) and MAPK signaling pathway (*p*=2.58E-09).

### 3.3. qRT-PCR Confirmation for PMOP

**Figure 4:**
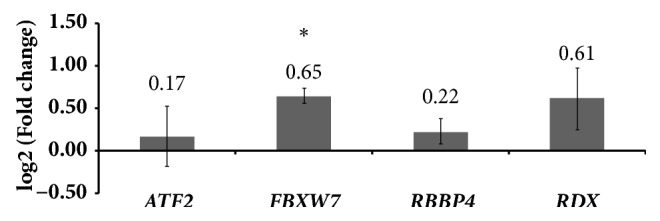
**qRT-PCR verification results for PMOP**. The X-axis indicated eight selected DEGs and the Y-axis indicated log 2 (fold change) for qRT-PCR results.

To manifest the expression of integrated analysis for PMOP, we compared the results of qRT-PCR and integrated analysis of four genes including ATF2, FBXW7, RBBP4, and RDX. In Figure 4, the expressions of ATF2, FBXW7, RDX, and RBBP4 were all consistent with our integrated analysis. These 4 selected DEGs for qRT-PCR confirmation were derived from the top 10 degrees. Based on previous studies, ATF2, FBXW7, and RDX were osteoporosis-related DEGs. Saul D [21] found that RBBP4 (involved in age-related memory loss) was increased in tibia callus after ovariectomy.

### 3.4. Coanalysis of PMOP versus Normal Control and LWDH Treatment versus PMOP

Combining the results of PMOP versus normal control and LWDH treatment versus PMOP, we obtained 58 common DEGs; among which, 43 genes were upregulated in PMOP versus normal control and downregulated in LWDH treatment versus PMOP; 15 genes were upregulated in LWDH treatment versus PMOP and downregulated in PMOP versus normal control. The details of these 58 common DEGs were shown in Table 1.

### 3.5. Functional Annotation of Common DEGs

In Supplementary Figure 1, several GO categories of up- and downregulated common DEGs were enriched in GO terms associated with regulation of biological process (*p*=9.84E-05) and biological regulation (*p*=0.000669575) in biological process (BP), intracellular part (*p*=0.003479728) and intracellular part (*p*=0.0023611) in cellular component (CC), and protein binding (*p*=0.00011324), enzyme binding (*p*=0.00052695), and catalytic activity (*p*=0.003357531) in molecular functions (MF). The most significantly different biological process (BP) of upregulated and downregulated DEGs was the regulation of biological process. And the most significantly different molecular functions (MF) were the protein binding.

### 3.6. PPI Network of Common DEGs

**Figure 5:**
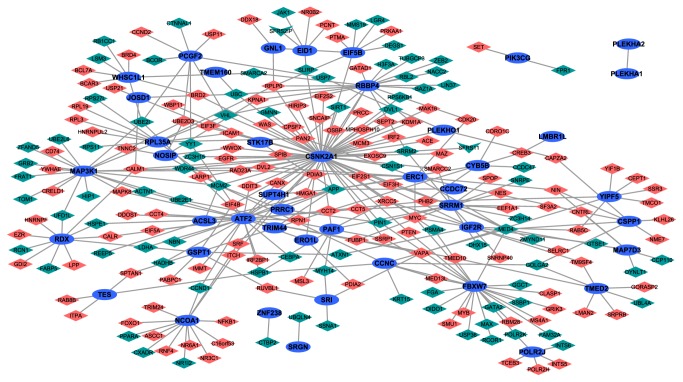
**Protein-protein network of 58 common DEGs**. All points were differentially expressed in both groups; the blue ovals represented the common and oppositely expressed genes; the diamonds represented the differentially expressed genes in PMOP versus normal control; the inverted triangle represents the differentially expressed genes in LWDH treatment versus PMOP, of which red represented upregulated mRNA and green represented downregulated mRNA.

To identify potential interactions between DEGs, a PPI network was constructed. The results identified 264 nodes (genes) and 347 edges of DEGs in Figure 5. All points were differentially expressed in both groups, with blue ovals representing the common and opposite genes, diamonds representing DEGs in PMOP versus normal control, and inverted triangles representing DEGs in LWDH treatment versus PMOP, of which red represents upregulated mRNA and green represents downregulated mRNA. Among them, the higher degrees of genes were CSNK2A1 (degree=66), ATF2 (degree=33), FBXW7 (degree=26), RBBP4 (degree=23), MAP3K1 (degree=20), SRRM1 (degree =19), NCOA1 (degree=15), RDX (degree=11), and APP (degree=10), among which, the hub proteins were CSNK2A1, ATF2, and FBXW7.

### 3.7. Predicted Gene Targets of LWDH

Using the BATMAN-TCM database, the potential mRNA targets of the six herbs of LWDH (ZE XIE, SHAN YAO, MU DAN PI, SHU DI HUANG, JIU YU ROU, and FU LING) were predicted. There were 177, 478, 73, 42, 0, and 503 targets for ZE XIE, SHAN YAO, MU DAN PI, SHU DI HUANG, JIU YU ROU, and FU LING, respectively. Among them, there were 10 common mRNA targets (ESR1, FGFR2, MED1, PGR, PRKCB, PTGS1, PTGS2, TRIM24, VDR, and WNT4) for the six herbs. Compared with the DEGs after treatment of LWDH, ATF2, FBXW7, and RDX were significantly differentially expressed in patients after treatment, and they are also the gene targets of LWDH. And, in the PPI network, ATF2, FBXW7, and RDX were among the genes which have high degree.

## 4. Discussion

Postmenopausal osteoporosis (PMOP) is a multifactorial chronic disease. With the increase of the elderly population, the incidence of osteoporosis is on the rise, which is a health issue of concern in the world [6]. Since the effective treatment is deficient, exploring the mechanism and finding novel therapeutic strategies for PMOP have long been an essential goal. In this study, we obtained 1732 DEGs between PMOP and normal controls by integrated microarray analysis.

Based on the function annotation analysis, we found that MAPK was a significantly enriched pathway for DEGs in PMOP. And previous study [22] has reported that MAPK signaling pathways are crucial in regulating osteogenesis, a genetic disorder affecting the bones. Lizhi Xing et al. [22] observed the upregulation of MAPK in the ovariectomy-induced bone loss. According to the functional annotation and PMOP-specific protein-protein interaction (PPI) network, four key DEGs (ATF2, FBXW7, RDX, and IGF2R) were speculated to be closely associated with PMOP.

ATF2 is a well-known osteoclastogenic transcription factor successfully identified by motif discovery analysis [23]. Osteoclast (also known as bone-resorbing cells) is a kind of bone tissue composition which is responsible for bone resorption. During bone resorption, the active osteoclasts secrete acidic substances and enzymes to break down the mineralized bone matrix and reduce the bone mass in this area [24]. In menopausal women, regulated by hormone, the activity of osteoclasts was accelerated so that the bone wall was thin. In our study, ATF2 is upregulated in PMOP group compared with normal controls, which supported previous studies. In addition, ATF2 is a hub protein of PMOP-specific PPI network which emphasized its importance in PMOP.

Radixin (RDX) is a cytoskeletal protein that may be important in linking actin to the plasma membrane. Among the ERM family members, only radixin is shown to be directly bound to the barbed ends of actin filaments. Previous studies have clarified the important roles of ERMs in various cellular processes such as epithelial morphogenesis, cell migration, and cell signal transduction [25]. Phosphorylated ERM protein plays an important role in cell growth, metabolism, and antiapoptosis and can promote cell migration and filamentous actin recombination and promote cell growth [26]. Upregulated RDX was found to be upregulated in PMOP compared with normal controls which were a potential regulator of PMOP.

FBXW7, an F-box protein component of the cullin-RING ubiquitin E3 ligase superfamilies, can recruit specific substrates to the RING E3 core through their variable carboxy-terminal protein-interaction domains. FBXW7 mediates the polyubiquitination and proteasomal degradation of active Notch. The study of Mi Yang [27] demonstrated that endogenous levels of FBXW7 protein were regulated by overexpression or inhibition of miR-497B195. In addition, overexpression of miR-497B195 was found to promote osteogenesis in endothelial cells of mice. In our study, FBXW7 is upregulated in PMOP group compared with normal controls which was speculated to involve with PMOP regulated by miR-497B195. Further research was needed to confirm this finding.

And previous studies evaluated the relationship between serum insulin-like growth factor-binding protein profiles and bone mineral density measurements in PMOP [28]. Insulin-like growth factor-binding protein, also known as IGFBP, serves as a carrier protein for insulin-like growth factor, which binds to insulin-like growth factor 2 receptor (IGF2R) [29]. The serum insulin-like growth factor-binding protein-2 ratio in postmenopausal women with osteoporosis was significantly higher (p < 0.02) than that in normal healthy postmenopausal women, but the serum insulin-like growth factor binding protein-3 ratio in women with osteoporosis was significantly lower (p < 0.01). Wuster et al. suggested that there is a positive correlation between bone mineral density and IGFBP-3 in postmenopausal women with osteoporosis [30].

Furthermore, three upregulated genes (ATF, RDX, and FBXW7) were found to be downregulated in patients with PMOP after treatment of LWDH in this present study. Based on the BATMAN-TCM database, the potential targets of four herbs of “LWDH (ZE XIE, SHAN YAO, MU DAN PI, SHU DI HUANG and FU LING)” were predicted. All the above three genes (ATF, RDB, and FBXW7) were predicted targets of LWDH which provided evidence for our finding.

## 5. Conclusion

Taken together, MAPK was found to be associated with PMOP. Four genes (ATF, RDB, FBXW7, and IGF2R) might be potential regulators and biomarkers of PMOP. Moreover, three genes (ATF, RDB, and FBXW7) were speculated to be closely associated with both the pathogenesis of PMOP and the therapeutic mechanism of LWDH for PMOP. Our finding contributed to providing clues for exploring mechanism and novel strategy of drug design for PMOP. However, small sample size of RT-PCR was a limitation for our study, and further research with large sample size was needed to confirm this conclusion.

## Figures and Tables

**Table 1:** Coexpressed and opposite trend of DEGs in the two groups.

**Id**	**Symbol**	**case vs normal**		**before vs after**	
		**Raw **P-Value	**Up-down**	**P**-**Value**	**Up-down**
2181	ACSL3	0.044463305	up	5.81E-05	down
23519	ANP32D	0.031671352	up	0.00020335	down
1386	ATF2	0.015288615	up	0.000105124	down
892	CCNC	0.037220624	up	4.23E-05	down
1370	CPN2	0.009788448	up	1.57E-05	down
1457	CSNK2A1	0.029560249	up	0.00035395	down
79848	CSPP1	0.011683224	up	1.11E-05	down
6374	CXCL5	0.027209429	up	6.23E-05	down
80777	CYB5B	0.000178896	up	0.000622171	down
23741	EID1	0.043185211	up	0.000455124	down
9669	EIF5B	0.014971999	up	0.00042694	down
23085	ERC1	0.032754729	up	0.000160588	down
30001	ERO1L	0.0154858	up	0.000625767	down
55294	FBXW7	0.037818674	up	0.000138841	down
2914	GRM4	0.023673494	up	0.000502142	down
2935	GSPT1	0.004988472	up	2.63E-05	down
3482	IGF2R	0.043016644	up	0.000342073	down
7850	IL1R2	0.025284329	up	3.93E-06	down
9929	JOSD1	0.013589301	up	0.000203472	down
51621	KLF13	0.020717689	up	7.15E-05	down
4214	MAP3K1	0.022243844	up	8.40E-05	down
79649	MAP7D3	0.003259237	up	0.000418951	down
158747	MOSPD2	0.001561148	up	0.000121954	down
7703	PCGF2	0.013528324	up	6.33E-05	down
5294	PIK3CG	0.001627058	up	0.00030047	down
59338	PLEKHA1	0.049102137	up	0.000452568	down
59339	PLEKHA2	0.007489418	up	1.26E-05	down
133619	PRRC1	0.022743976	up	0.00052731	down
5928	RBBP4	0.00010116	up	4.70E-05	down
5962	RDX	0.049953292	up	0.000486787	down
55680	RUFY2	0.001194843	up	0.000266002	down
5552	SRGN	0.009208736	up	0.000209717	down
6717	SRI	0.037181628	up	0.000508307	down
10250	SRRM1	0.000952409	up	6.51E-05	down
9262	STK17B	0.016461631	up	3.91E-05	down
26136	TES	0.000594943	up	0.000259901	down
10959	TMED2	0.046585899	up	0.000567562	down
54765	TRIM44	0.000191712	up	0.000565748	down
389898	UBE2NL	0.013171719	up	0.00048327	down
54904	WHSC1L1	0.020186972	up	1.24E-05	down
81555	YIPF5	0.018233854	up	0.000196894	down
10472	ZNF238	0.002670727	up	7.55E-05	down
7639	ZNF85	0.028282272	up	0.000212913	down
55811	ADCY10	0.012001458	down	0.000502024	up
55009	C19orf24	0.006292312	down	0.000610074	up
51372	CCDC72	0.000667113	down	0.00060282	up
2323	FLT3LG	0.017012385	down	0.000637474	up
2794	GNL1	0.012495179	down	0.000573231	up
55716	LMBR1L	0.001157605	down	0.000600187	up
8648	NCOA1	0.016190136	down	0.000192176	up
51070	NOSIP	0.008546637	down	0.000285444	up
54623	PAF1	0.034489911	down	0.000236501	up
51177	PLEKHO1	0.036853037	down	0.000331093	up
5439	POLR2J	0.026864334	down	0.000213443	up
6165	RPL35A	0.041712982	down	0.00055368	up
6827	SUPT4H1	0.016431549	down	0.000265733	up
54958	TMEM160	0.022504594	down	0.000255735	up
389136	VGLL3	0.049059686	down	0.000604032	up

## Data Availability

The data used to support the findings of this study are available from the corresponding author upon request.

## Supplementary Materials

Supplementary Materials
** Supplementary Figure 1. GO functional enrichment of 58 common DEGs**. A: biological progress; B: cellular component; and C: molecular function. The y-axis represented GO terms and the x-axis shows represented counts of DEmRNAs enriched in GO terms.** Supplementary Table **1. The primers of selected mRNAs (ATF2, FBXW7, RBBP4, and RDX).** Supplementary Table **2. The clinical features of the enrolled subjects from four datasets (GSE100609, GSE56815, GSE13850, and GSE7429).
