# Corrigendum to “Bioinformatics Analysis Reveals the Altered Gene Expression of Patients with Postmenopausal Osteoporosis Using Liuweidihuang Pills Treatment”

**DOI:** 10.1155/2019/6897187

**Published:** 2019-11-20

**Authors:** Rui Gong, Shan Ren, Menghui Chen, Yanli Wang, Guoliang Zhang, Lijuan Shi, Cuizhao Zhang, Ruihong Su, Yukun Li

**Affiliations:** ^1^Hebei Medical University Endocrine Teaching and Research Section, China; ^2^Department of Gerontology, The Third Hospital of Shijiazhuang, China; ^3^Department of Endocrinology, Third Affiliated Hospital of Hebei Medical University, China; ^4^Department of ICU, Hebei General Hospital, China; ^5^Department of Cardiothoracic Surgery, The Third Hospital of Shijiazhuang, China; ^6^Obstetrics and Gynecology, The Third Hospital of Shijiazhuang, China; ^7^Department of Endocrinology, The Third Hospital of Shijiazhuang, China; ^8^Department of Laboratory, The Third Hospital of Shijiazhuang, China

In article titled “Bioinformatics Analysis Reveals the Altered Gene Expression of Patients with Postmenopausal Osteoporosis Using Liuweidihuang Pills Treatment” [1], there was an error in the affiliation details of Rui Gong and Yukun Li as shown below.

Rui Gong was only affiliated to affiliation number 1 “Hebei Medical University Endocrine Research Institute.” However, they should be affiliated to affiliations 1, 2, and 3.

Yukun Li was wrongly affiliated to affiliation number 2 “Department of Gerontology, The Third Hospital of Shijiazhuang, China.” However, they should be affiliated to affiliation number 3 “Department of Endocrinology, Third Affiliated Hospital of Hebei Medical University, China.”

The corrected authors' list and affiliations are shown above, and it has been added in-line to the published article.
