# Supplementary material for: Bioinformatics Analysis Reveals the Altered Gene Expression of Patients with Postmenopausal Osteoporosis Using Liuweidihuang Pills Treatment
Source: Biomed Res Int. 2019 Jan 27;2019:1907906. doi: 10.1155/2019/1907906 (PMC6369488; doi:10.1155/2019/1907906)
Supplement: Supplementary Materials — Supplementary Figure 1. GO functional enrichment of 58 common DEGs. A: biological progress; B: cellular component; and C: molecular function. The y-axis represented GO terms and the x-axis shows represented counts of DEmRNAs enriched in GO terms. Supplementary Table 1. The primers of selected mRNAs (ATF2, FBXW7, RBBP4, and RDX). Supplementary Table 2. The clinical features of the enrolled subjects from four datasets (GSE100609, GSE56815, GSE13850, and GSE7429). [file 1907906.f1.pdf]

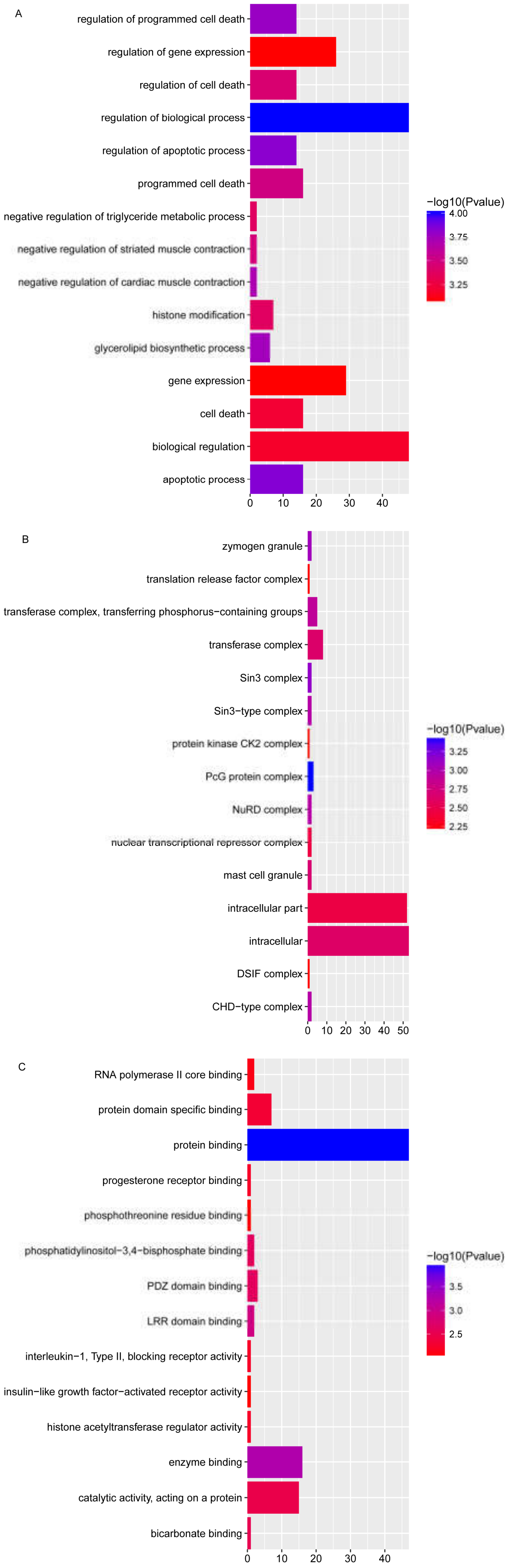

Supplementry Figure 1

Supplementary Table 1 The primers of selected mRNAs

**Primer name**	**Sequence of primer(5'to3')**	**Size of production （bp）**
GAPDH-F (endogenous control)	GGAGCGAGATCCCTCCAAAAT	197
GAPDH-R (endogenous control)	GGCTGTTGTCATACTTCTCATGG	
ATF, 2-F	TTTGACCCAGCAACATCCTCC	168
ATF2-R	TGGGTCTGTGGAGTTGTGTGA	
FBXW7-F	GTCCCGAGAAGCGGTTTGATA	100
FBXW7-R	TGCTCAGGCACGTCAGAAAAG	
RBBP4-F	TCAGTTTGATGCGTCACACTAC	150
RBBP4-R	GCGATGATACAAGGGTTCTGG	
RDX-F	GCCCAAGCCAGAGATGAAACC	166
RDX-R	ATGCATACAGTTTGGGTCCCC	

Supplementary table 2 The clinical features of the enrolled subjects

**GSE7429**			
GEO accession	Sample title	Sample characteristics 1	Sample characteristics 2
GSM179758	Bcell-highBMD-1	postmenopause, age=57	-
GSM179759	Bcell-highBMD-2	postmenopause, age=57	-
GSM179760	Bcell-highBMD-3	postmenopause, age=60	-
GSM179761	Bcell-highBMD-4	postmenopause, age=58	-
GSM179762	Bcell-highBMD-5	postmenopause, age=54	-
GSM179763	Bcell-highBMD-6	postmenopause, age=54	-
GSM179764	Bcell-highBMD-7	postmenopause, age=58	-
GSM179765	Bcell-highBMD-8	postmenopause, age=58	-
GSM179766	Bcell-highBMD-9	postmenopause, age=55	-
GSM179767	Bcell-highBMD-10	postmenopause, age=58	-
GSM179768	Bcell-lowBMD-1	postmenopause, age=58	-
GSM179769	Bcell-lowBMD-2	postmenopause, age=56	-
GSM179770	Bcell-lowBMD-3	postmenopause, age=58	-
GSM179771	Bcell-lowBMD-4	postmenopause, age=59	-
GSM179772	Bcell-lowBMD-5	postmenopause, age=58	-
GSM179773	Bcell-lowBMD-6	postmenopause, age=60	-
GSM179774	Bcell-lowBMD-7	postmenopause, age=59	-
GSM179775	Bcell-lowBMD-8	postmenopause, age=56	-
GSM179776	Bcell-lowBMD-9	postmenopause, age=59	-
GSM179777	Bcell-lowBMD-10	postmenopause, age=56	-

GSE13850			
GEO accession	Sample title	Sample characteristics 1	Sample characteristics 2
GSM179758	Bcell-highBMD-1	postmenopause, age=57	-
GSM179759	Bcell-highBMD-2	postmenopause, age=57	-
GSM179760	Bcell-highBMD-3	postmenopause, age=60	-
GSM179761	Bcell-highBMD-4	postmenopause, age=58	-
GSM179762	Bcell-highBMD-5	postmenopause, age=54	-
GSM179763	Bcell-highBMD-6	postmenopause, age=54	-
GSM179764	Bcell-highBMD-7	postmenopause, age=58	-
GSM179765	Bcell-highBMD-8	postmenopause, age=58	-
GSM179766	Bcell-highBMD-9	postmenopause, age=55	-
GSM179767	Bcell-highBMD-10	postmenopause, age=58	-
GSM179768	Bcell-lowBMD-1	postmenopause, age=58	-
GSM179769	Bcell-lowBMD-2	postmenopause, age=56	-
GSM179770	Bcell-lowBMD-3	postmenopause, age=58	-
GSM179771	Bcell-lowBMD-4	postmenopause, age=59	-
GSM179772	Bcell-lowBMD-5	postmenopause, age=58	-
GSM179773	Bcell-lowBMD-6	postmenopause, age=60	-
GSM179774	Bcell-lowBMD-7	postmenopause, age=59	-
GSM179775	Bcell-lowBMD-8	postmenopause, age=56	-
GSM179776	Bcell-lowBMD-9	postmenopause, age=59	-
GSM179777	Bcell-lowBMD-10	postmenopause, age=56	-
GSM348199	B cells_Postmenopausal_Smoking_high BMD_rep1	Postmenopause, smoking	-
GSM348200	B cells_Postmenopausal_Smoking_high BMD_rep2	Postmenopause, smoking	-
GSM348201	B cells_Postmenopausal_Smoking_high BMD_rep3	Postmenopause, smoking	-
GSM348202	B cells_Postmenopausal_Smoking_high BMD_rep4	Postmenopause, smoking	-
GSM348203	B cells_Postmenopausal_Smoking_high BMD_rep5	Postmenopause, smoking	-
GSM348204	B cells_Postmenopausal_Smoking_high BMD_rep6	Postmenopause, smoking	-
GSM348205	B cells_Postmenopausal_Smoking_high BMD_rep7	Postmenopause, smoking	-
GSM348206	B cells_Postmenopausal_Smoking_high BMD_rep8	Postmenopause, smoking	-
GSM348207	B cells_Postmenopausal_Smoking_high BMD_rep9	Postmenopause, smoking	-
GSM348208	B cells_Postmenopausal_Smoking_high BMD_rep10	Postmenopause, smoking	-
GSM348209	B cells_Postmenopausal_Smoking_high BMD_rep1	Postmenopause, smoking	-

GSM348210	B cells_Postmenopausal_Smoking_high BMD_rep2	Postmenopause, smoking	-
GSM348211	B cells_Postmenopausal_Smoking_high BMD_rep3	Postmenopause, smoking	-
GSM348212	B cells_Postmenopausal_Smoking_high BMD_rep4	Postmenopause, smoking	-
GSM348213	B cells_Postmenopausal_Smoking_high BMD_rep5	Postmenopause, smoking	-
GSM348214	B cells_Postmenopausal_Smoking_high BMD_rep6	Postmenopause, smoking	-
GSM348215	B cells_Postmenopausal_Smoking_high BMD_rep7	Postmenopause, smoking	-
GSM348216	B cells_Postmenopausal_Smoking_high BMD_rep8	Postmenopause, smoking	-
GSM348217	B cells_Postmenopausal_Smoking_high BMD_rep9	Postmenopause, smoking	-
GSM348218	B cells_Postmenopausal_Smoking_high BMD_rep10	Postmenopause, smoking	-

GSE56815

GEO accession	Sample title	Sample characteristics 1	Sample characteristics 2
GSM1369756	Monocytes from a postmonopausal high BMD woman (H-GE-01M.CEL)	gender: Female	bone mineral density: high BMD
GSM1369757	Monocytes from a postmonopausal high BMD woman (H-GE-04M.CEL)	gender: Female	bone mineral density: high BMD
GSM1369758	Monocytes from a postmonopausal high BMD woman (H-GE-06M.CEL)	gender: Female	bone mineral density: high BMD
GSM1369759	Monocytes from a postmonopausal high BMD woman (H-GE-07M.CEL)	gender: Female	bone mineral density: high BMD
GSM1369760	Monocytes from a postmonopausal high BMD woman (H-GE-10M.CEL)	gender: Female	bone mineral density: high BMD
GSM1369761	Monocytes from a postmonopausal high BMD woman (H-GE-11M.CEL)	gender: Female	bone mineral density: high BMD
GSM1369762	Monocytes from a postmonopausal high BMD woman (H-GE-13M.CEL)	gender: Female	bone mineral density: high BMD
GSM1369765	Monocytes from a postmonopausal high BMD woman (H-GE-18M.CEL)	gender: Female	bone mineral density: high BMD
GSM1369767	Monocytes from a postmonopausal high BMD woman (H-GE-20M.CEL)	gender: Female	bone mineral density: high BMD
GSM1369769	Monocytes from a postmonopausal high BMD woman (H-GE-23M.CEL)	gender: Female	bone mineral density: high BMD
GSM1369771	Monocytes from a postmonopausal high BMD woman (H-GE-26M.CEL)	gender: Female	bone mineral density: high BMD
GSM1369777	Monocytes from a postmonopausal high BMD woman (H-GE-34M.CEL)	gender: Female	bone mineral density: high BMD

GSM1369778	Monocytes from a postmonopausal high BMD woman (H-GE-42M.CEL)	gender: Female	bone mineral density: high BMD
GSM1369779	Monocytes from a postmonopausal high BMD woman (H-GE-45M.CEL)	gender: Female	bone mineral density: high BMD
GSM1369780	Monocytes from a postmonopausal high BMD woman (H-GE-47M.CEL)	gender: Female	bone mineral density: high BMD
GSM1369781	Monocytes from a postmonopausal high BMD woman (H-GE-53M.CEL)	gender: Female	bone mineral density: high BMD
GSM1369782	Monocytes from a postmonopausal high BMD woman (H-GE-55M.CEL)	gender: Female	bone mineral density: high BMD
GSM1369783	Monocytes from a postmonopausal high BMD woman (H-GE-59M.CEL)	gender: Female	bone mineral density: high BMD
GSM1369784	Monocytes from a postmonopausal high BMD woman (H-GE-65M.CEL)	gender: Female	bone mineral density: high BMD
GSM1369792	Monocytes from a postmonopausal high BMD woman (H-GE-80M.CEL)	gender: Female	bone mineral density: high BMD
GSM1369797	Monocytes from a postmonopausal low BMD woman (L-GE-25M.CEL)	gender: Female	bone mineral density: low BMD
GSM1369798	Monocytes from a postmonopausal low BMD woman (L-GE-31M.CEL)	gender: Female	bone mineral density: low BMD
GSM1369799	Monocytes from a postmonopausal low BMD woman (L-GE-32M.CEL)	gender: Female	bone mineral density: low BMD
GSM1369800	Monocytes from a postmonopausal low BMD woman (L-GE-35M.CEL)	gender: Female	bone mineral density: low BMD
GSM1369802	Monocytes from a postmonopausal low BMD woman (L-GE-37M.CEL)	gender: Female	bone mineral density: low BMD
GSM1369803	Monocytes from a postmonopausal low BMD woman (L-GE-38M.CEL)	gender: Female	bone mineral density: low BMD
GSM1369805	Monocytes from a postmonopausal low BMD woman (L-GE-40M.CEL)	gender: Female	bone mineral density: low BMD
GSM1369807	Monocytes from a postmonopausal low BMD woman (L-GE-43M.CEL)	gender: Female	bone mineral density: low BMD
GSM1369810	Monocytes from a postmonopausal low BMD woman (L-GE-48M.CEL)	gender: Female	bone mineral density: low BMD
GSM1369812	Monocytes from a postmonopausal low BMD woman (L-GE-50M.CEL)	gender: Female	bone mineral density: low BMD
GSM1369813	Monocytes from a postmonopausal low BMD woman (L-GE-51M.CEL)	gender: Female	bone mineral density: low BMD
GSM1369814	Monocytes from a postmonopausal low BMD woman (L-GE-52M.CEL)	gender: Female	bone mineral density: low BMD
GSM1369816	Monocytes from a postmonopausal low BMD woman (L-GE-56M.CEL)	gender: Female	bone mineral density: low BMD
GSM1369821	Monocytes from a postmonopausal low BMD woman (L-GE-62M.CEL)	gender: Female	bone mineral density: low BMD

GSM1369822	Monocytes from a postmonopausal low BMD woman (L-GE-63M.CEL)	gender: Female	bone mineral density: low BMD
GSM1369823	Monocytes from a postmonopausal low BMD woman (L-GE-64M.CEL)	gender: Female	bone mineral density: low BMD
GSM1369824	Monocytes from a postmonopausal low BMD woman (L-GE-66M.CEL)	gender: Female	bone mineral density: low BMD
GSM1369825	Monocytes from a postmonopausal low BMD woman (L-GE-67M.CEL)	gender: Female	bone mineral density: low BMD
GSM1369826	Monocytes from a postmonopausal low BMD woman (L-GE-69M.CEL)	gender: Female	bone mineral density: low BMD
GSM1369828	Monocytes from a postmonopausal low BMD woman (L-GE-72M.CEL)	gender: Female	bone mineral density: low BMD

GSE100609

GEO accession	Sample title	Sample characteristics 1	Sample characteristics 2
GSM179758	Bcell-highBMD-1	postmenopause, age=57	-
GSM179759	Bcell-highBMD-2	postmenopause, age=57	-
GSM179760	Bcell-highBMD-3	postmenopause, age=60	-
GSM179761	Bcell-highBMD-4	postmenopause, age=58	-
GSM179762	Bcell-highBMD-5	postmenopause, age=54	-
GSM179763	Bcell-highBMD-6	postmenopause, age=54	-
GSM179764	Bcell-highBMD-7	postmenopause, age=58	-
GSM179765	Bcell-highBMD-8	postmenopause, age=58	-

!Sample_geo_acc	!Sample_title	!Sample_characteristics_ch1
GSM179758	Bcell-highBMD-1	postmenopause, age=57,Spine Z-score=2.28,Hip Z-score=1.46
GSM179759	Bcell-highBMD-2	postmenopause, age=57,Spine Z-score=2.37,Hip Z-score=0.97
GSM179760	Bcell-highBMD-3	postmenopause, age=57,Spine Z-score=1.66,Hip Z-score=1.29
GSM179761	Bcell-highBMD-4	postmenopause, age=60,Spine Z-score=1.91,Hip Z-score=2.41
GSM179762	Bcell-highBMD-5	postmenopause, age=58,Spine Z-score=1.23,Hip Z-score=0.80
GSM179763	Bcell-highBMD-6	postmenopause, age=54,Spine Z-score=2.09,Hip Z-score=3.07
GSM179764	Bcell-highBMD-7	postmenopause, age=54,Spine Z-score=2.25,Hip Z-score=2.56
GSM179765	Bcell-highBMD-8	postmenopause, age=58,Spine Z-score=1.84,Hip Z-score=0.57
GSM179766	Bcell-highBMD-9	postmenopause, age=55,Spine Z-score=2.74,Hip Z-score=1.19
GSM179767	Bcell-highBMD-10	postmenopause, age=58,Spine Z-score=1.94,Hip Z-score=1.93
GSM179768	Bcell-lowBMD-1	postmenopause, age=58,Spine Z-score=-0.9,Hip Z-score=-0.77
GSM179769	Bcell-lowBMD-2	postmenopause, age=56,Spine Z-score=-1.62,Hip Z-score=-1.15
GSM179770	Bcell-lowBMD-3	postmenopause, age=58,Spine Z-score=-1.3,Hip Z-score=-1.16
GSM179771	Bcell-lowBMD-4	postmenopause, age=59,Spine Z-score=0.16,Hip Z-score=-0.9
GSM179772	Bcell-lowBMD-5	postmenopause, age=58,Spine Z-score=-0.7,Hip Z-score=-0.47
GSM179773	Bcell-lowBMD-6	postmenopause, age=60,Spine Z-score=-0.2,Hip Z-score=-0.88
GSM179774	Bcell-lowBMD-7	postmenopause, age=59,Spine Z-score=-0.52,Hip Z-score=-0.89
GSM179775	Bcell-lowBMD-8	postmenopause, age=56,Spine Z-score=0.3,Hip Z-score=-0.61
GSM179776	Bcell-lowBMD-9	postmenopause, age=59,Spine Z-score=-2.26,Hip Z-score=-1.31
GSM179777	Bcell-lowBMD-10	postmenopause, age=56,Spine Z-score=-0.73,Hip Z-score=-0.65
